# Addition of a combination of creatine, carnitine, and choline to a commercial diet increases postprandial plasma creatine and creatinine concentrations in adult dogs

**DOI:** 10.3389/fvets.2022.1063169

**Published:** 2022-11-25

**Authors:** Sydney Banton, Ulrike Braun, E. James Squires, Anna K. Shoveller

**Affiliations:** ^1^Department of Animal Biosciences, University of Guelph, Guelph, ON, Canada; ^2^AlzChem Trostberg GmbH, Trostberg, Germany

**Keywords:** creatine, creatinine, dogs, meal response, amino acid metabolism

## Abstract

Creatine is a nitrogenous compound essential for cellular energy homeostasis found in animal protein; however, when heat-processed for pet food, creatine is degraded to creatinine, which is not metabolically active and excreted in urine. The objective of the present investigation was to define the postprandial plasma creatine and creatinine response in dogs fed a commercial diet (CON) formulated for adult dogs, top-dressed with a combination of creatine (9.6 g/kg dry matter, DM), carnitine (2.13 g/kg DM) and choline (0.24 g/kg DM; CCC), methionine (2.6 g/kg DM; MET), or taurine (0.7 g/kg DM; TAU). Eight adult Beagles were fed one of the four diets for 7 days in a Latin Square design with no washout period. On day 7, cephalic catheters were placed and blood samples were collected before being fed (fasted) and up to 6 h post-meal. Creatine and creatinine were analyzed using HPLC and data analyzed using PROC GLIMMIX in SAS. Plasma creatine concentrations were higher in dogs fed CCC (103 ± 10 μmol/L) compared to MET (72 ± 7 μmol/L) at fasted (*P* < 0.05) and higher compared to all other treatments from 15 to 360 min post-meal (*P* < 0.05). Plasma creatinine concentrations were higher in dogs fed CCC from 60 to 180 min compared to all other treatments. These data suggest that when creatine, carnitine and choline are top-dressed for 7 days, plasma creatine is rapidly absorbed and remains elevated up to 6 h post-meal. This may have implications for energy metabolism and should be considered when using creatinine as a diagnostic tool in dogs.

## Introduction

Creatine, an essential nitrogenous compound, is synthesized from the amino acids glycine and arginine to produce ornithine and guanidinoacetate (GAA). Once synthesized, GAA can be methylated by S-adenosylmethionine (SAM) to creatine and S-adenosylhomocysteine, which is quickly metabolized to homocysteine. This reaction uses more methyl groups from SAM than any other methylation reaction (1). Creatine is stored in high concentrations in both skeletal and cardiac muscle as both creatine and phosphocreatine, which is available for immediate regeneration of ATP during short bouts of intense exercise [reviewed in (2)]. More specifically, during initial intense exercise, phosphocreatine is readily and quickly used for ATP regeneration whereas aerobic metabolism provides a significant amount of ATP during subsequent bouts of intense exercise (3). Creatine may also play a role in modulating neurological, neuromuscular and atherosclerotic disease [reviewed in (4)].

Creatine synthesis has the potential to present a burden for amino acid metabolism as it involves three amino acid precursors, glycine, arginine and methionine, the precursor to SAM. In fact, some studies in rats supplemented with creatine report greater plasma glycine concentrations (5) and lower plasma homocysteine concentrations (5, 6). Similar to creatine, carnitine is a major methyl acceptor and endogenous metabolite of methionine that plays a role in generating energy *via* fatty acid transport into the mitochondria (7). On the other hand, choline can act as a methyl donor in the remethylation pathway to generate methionine from homocysteine.

In vertebrates, creatine is irreversibly and non-enzymatically dehydrated to produce creatinine at a rate of about 1.5–2% of the total creatine pool per day (8) and, as such, serum and urine creatinine is used as a measure of glomerular filtration rate in dogs to clinically assess kidney disease. Although no reference range exists for plasma or serum creatine in dogs, several healthy reference ranges have been defined for serum creatinine in dogs. Extremes in serum creatinine have been reported from 35 to 250 μmol/L but reference ranges can depend on the laboratory method used and on the breed of dog (9). In fact, a separate reference range has been defined for greyhound dogs from 106 to 168 μmol/L given that creatinine concentrations are directly related to muscle mass (10). Accordingly, this reference range is higher than the reference range determined in the same study for healthy mixed breed dogs [70–150 μmol/L; (10)]. The serum concentrations of creatinine need to be interpreted carefully with an understanding of dietary composition and total feed intake, the body composition of the individual, and the intensity and duration of exercise that the individual participates in.

Sources of creatine in pet food are animal tissues and by-products; however, after heat-processing, creatine in kibble and meat and bone meal is rapidly degraded to creatinine resulting in lower concentrations of creatine and higher concentrations of creatinine in cooked pet food compared to raw meats (11). Although not currently supplemented, or even commonly measured in pet food, creatine has important implications for amino acid and energy metabolism in the dog. As such, the objective of the current study was to define the postprandial plasma creatine and creatinine response in dogs fed a commercial meat-based diet top-dressed with methyl acceptors, creatine and carnitine, and methyl donor, choline, or methionine, or a downstream metabolite of methionine, taurine. We hypothesize that dogs supplemented with creatine, carnitine and choline will have greater postprandial plasma creatine and creatinine concentrations compared to dogs fed methionine, taurine and control.

## Materials and methods

### Animals, dietary treatments, and meal response

All data that will be presented herein were collected but not published in Banton et al. (12) and as such, a detailed explanation of the methods can be found there. In short, eight pair-housed healthy Beagle dogs (1.6 ± 0.04 yrs, 7.8 ± 1.5 kg) were fed a commercial control diet formulated for all life stages and fed to maintain body weight (BW; Nutrience Grain-Free Pork, Lamb and Duck Formula, single batch, Rolf C. Hagen Inc., QC, Canada, Supplementary Table 1). This diet was fed either on its own (CON) or supplemented with 2.6 g/kg DM 99% DL-methionine (MET) to achieve 2.2 times the National Research Council (13) recommended allowance for adult dogs (7.2 g/kg dry matter; DM); 0.7 g/kg DM 98.5% taurine (TAU) to achieve a similar amount to a taurine supplemented diet for dogs with early cardiac disease (Royal Canin Early Cardiac Dry Dog Food, Mars Pet Care, St. Charles, MO; 1.6 g/kg DM); or a mixture of 9.6 g/kg DM 99.5% creatine monohydrate, 2.13 g/kg DM 60% choline chloride and 0.24 g/kg DM 50% L-carnitine (CCC). The control diet contained 0.195 g/kg of creatine and 0.563 g/kg creatinine on an as-fed basis. After supplementation, dogs fed CCC received a total of 9.123 g/kg creatine on an as-fed basis. The creatine dose was selected based on McBreairty et al. (14) study done in pigs, which is 200 mg/kg BW/day and is similar to a typical creatine dosing level in humans (15). Choline was supplemented to achieve 2.3 times the NRC recommended allowance for adult dogs (3.8 g/kg DM) and carnitine was supplemented to exceed the commonly supplemented dose of 0.1–0.2 g/kg DM in dog food [0.33 g/kg DM; (16)]. Dogs were fed one of the treatments or CON once daily for 7 d in a complete, randomized 4 × 4 Latin Square design. On d 7, a cephalic catheter was placed, a fasted blood sample was taken, the meal was fed and blood samples were collected at 15, 30, 60, 90, 120, 180, 240, 300, and 360 min after the meal. Blood was placed in a sodium-heparin tube and centrifuged at 4°C at 1,200 × *g* for 10 min. Plasma was separated and stored at −80°C until analysis.

### Creatine and creatinine analysis

The creatine and creatinine content of the control diet was analyzed at AlzChem Trostberg GmbH (Trostberg, Germany) using an ion chromatography system (Dionex ICS3000 or 5000) as reported in van der Poel et al. (17). The creatine and creatinine concentrations in the plasma was analyzed using High Performance Liquid Chromatography (HPLC) [Adapted from (18); Aligent Technologies, Santa Clara, CA]. Briefly, 60 uL of trifluoroacetic acid (TFA) was added to 300 uL of plasma and 300 uL of tris buffer in order to deproteinate the plasma. The sample was set on ice for 10 min before centrifuging at 14,500 rpm for 10 min. The supernatant was then filtered through a 0.2 uM nylon filter. Creatine and Creatinine (100 uL injection volume) were separated in a Hypercarb column (4.6 × 100 mm, 5 um; Fisher Scientific, Ottawa, ON) that was maintained at room temperature using HPLC with UV detection (210 nm) with a mobile phase of 3% acetonitrile and 0.1% TFA and a flow rate of 0.8 mL/min. Creatine and creatinine peaks were compared with known standards (Creatine Monohydrate, 99% and Anhydrous Creatinine, 98% from Sigma-Aldrich, St. Louis, MO).

### Statistical analysis

Concentrations of plasma creatine and creatinine were analyzed as repeated measures using the PROC GLIMMIX procedure in SAS (SAS version 9.4, SAS Inst., Inc., Cary, NC). Dietary treatment and time were treated as fixed effects and period and dog were treated as random effects. In the statistical model, the effect of dietary treatment, time, and their interaction was evaluated. For each variable, model assumptions were assessed through residual analysis. Residuals for creatine were not normally distributed and as such, data were log-transformed for analysis. Means were separated using the Tukey–Kramer adjustment, and results were deemed significant at *P* ≤ 0.05 and trends at 0.05 < *P* ≤ 0.10.

## Results

Residuals for creatine were not homogenous after log-transformation of the data but they were normally distributed. Although the homogeneity assumption of the model was not met, this is likely due to the much larger variation in plasma creatine concentrations in dogs fed CCC compared to the other treatment groups, which was expected since these dogs were supplemented with creatine.

**Figure 1:**
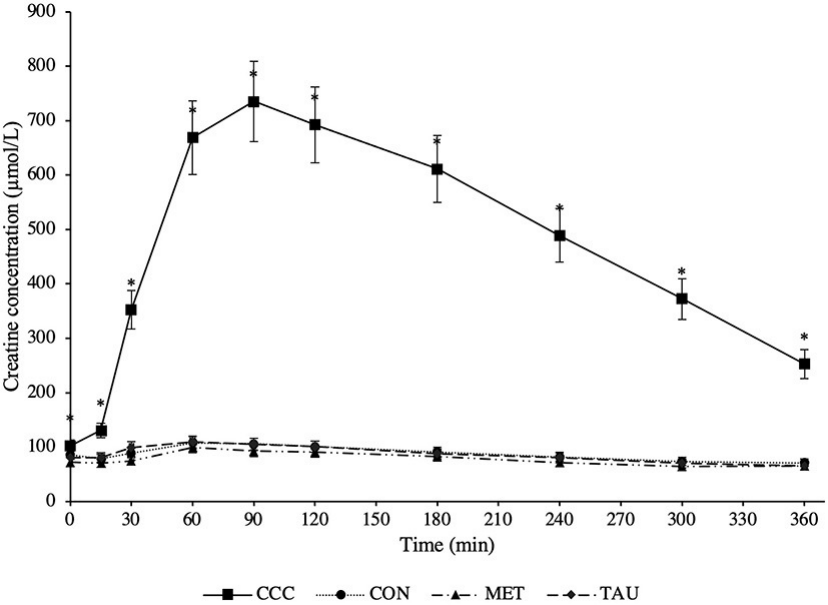
Mean plasma creatine concentrations (μmol/L) in dogs from fasted (0 min) to 360 min post-meal on either creatine, carnitine and choline (CCC), control (CON), methionine (MET), or taurine (TAU) supplementation. Values are presented as least squares means (lsmeans) ± SEM. The asterisk indicates a significant time by treatment interaction effect (*P* < 0.05) at each time point.

**Figure 2:**
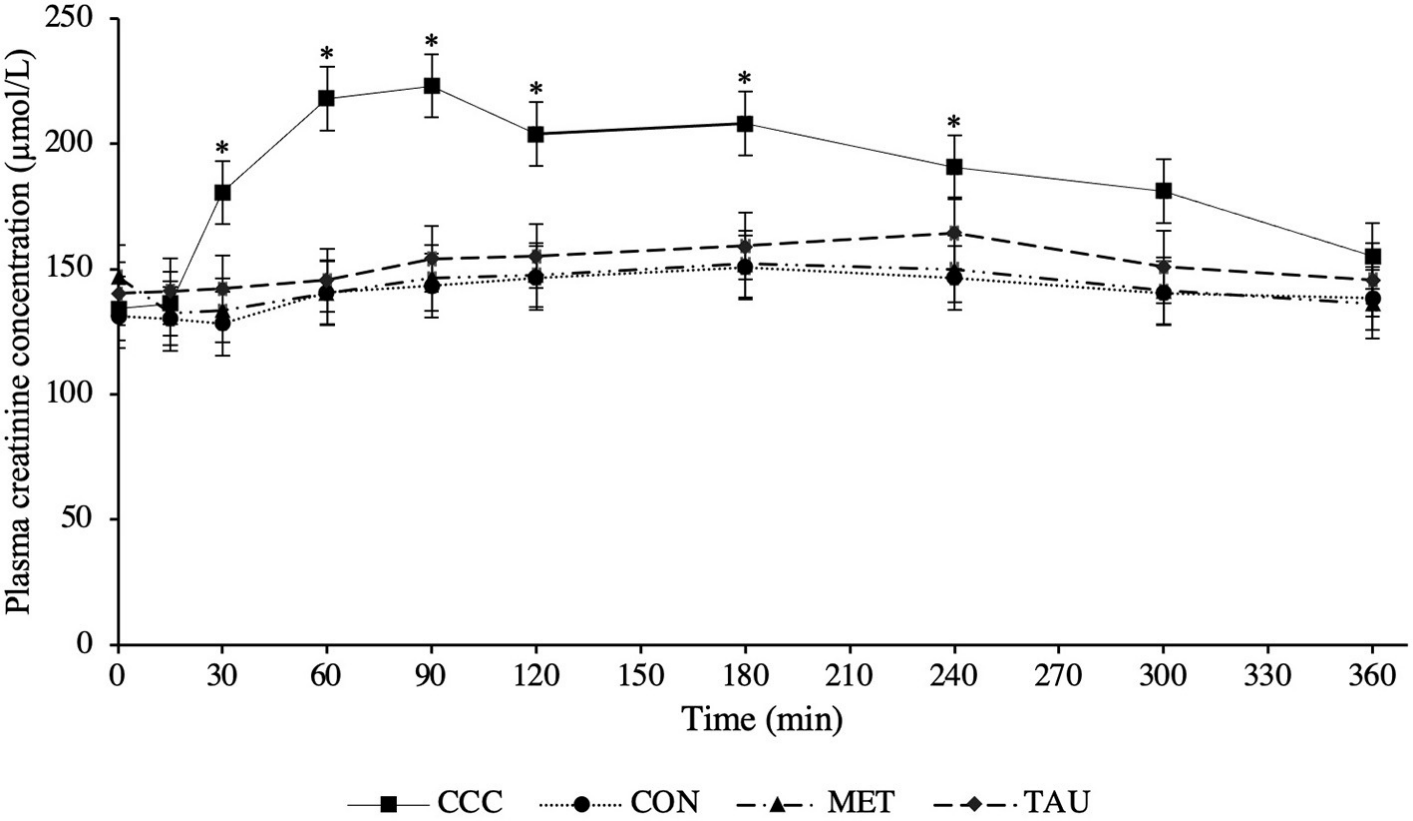
Mean plasma creatinine concentrations (μmol/L) in dogs from fasted (0 min) to 360 min post-meal on either creatine, carnitine and choline (CCC), control (CON), methionine (MET), or taurine (TAU) supplementation. Values are presented as least squares means (lsmeans) ± SEM. The asterisk indicates a significant time by treatment interaction effect (*P* < 0.05) at each time point.

There was a significant time by treatment interaction effect for both plasma creatine (*P* < 0.0001) and creatinine (*P* < 0.0001; Figures 1, 2, respectively). Fasted plasma creatine concentrations were higher in dogs fed CCC (103 ± 10 μmol/L) compared to dogs fed MET (72 ± 7 μmol/L; *P* = 0.0347), but similar to dogs fed CON and TAU. In addition, plasma creatine concentrations were higher in dogs fed CCC compared to all other treatments from 15 to 360 min post-meal (*P* < 0.05). Plasma creatinine concentrations were higher in dogs fed CCC compared to dogs fed CON (*P* = 0.0117) and MET (*P* = 0.0285) at 30 min post-meal and higher in dogs fed CCC from 60 to 180 min post-meal compared to all other treatments (*P* < 0.05). In addition, plasma creatinine was higher in dogs fed CCC compared to CON (*P* = 0.0458) and tended to be higher compared to dogs fed MET (*P* = 0.0861) at 240 min post-meal. Plasma creatinine concentrations tended to be higher in dogs fed CCC compared to dogs fed CON (*P* = 0.0779) and MET (*P* = 0.0994) at 300 min post-meal.

As dogs on MET, TAU and CON did not differ in their plasma creatine or creatinine concentrations at time 0 (fasted), a reference range for this population of dogs was calculated using pooled data across these three treatments (*n* = 24). Fasted plasma creatine ranged from 45.8 to 137.0 μmol/L with an average concentration of 83.0 ± 24.7 μmol/L, therefore the reference range was determined to be 33.6–132.4 μmol/L (mean ± 2 SDs). Fasted plasma creatinine ranged from 62.7 to 182.3 μmol/L with an average concentration of 139.4 ± 28.1 μmol/L, therefore the reference range was determined to be 83.3–195.5 μmol/L.

## Discussion

As hypothesized, creatine concentrations increased following the meal proving effective dosing in the CCC treatment and agreeing with previous reports. Harris et al. (19) reported peak creatine concentrations that were 4 times higher than fasted at 3 h post-meal in Beagles supplemented with creatine monohydrate at ~167 mg/kg after only 1 day. Lowe et al. (20) reported peak creatine concentrations that were 4.5 or 5.7 times higher than fasted at 1 and 2 h post-meal in Beagles supplemented with creatine monohydrate at either 200 or 400 mg/kg BW/day, respectively for 28 days. In the present study where we supplied creatine at 200 mg/kg BW/d, creatine reached peak concentrations that were seven times higher than the control diet after 90 min and remained elevated in plasma until at least 6 h post-meal. Therefore, when creatine monohydrate is top-dressed, it is rapidly absorbed and remains elevated in circulating plasma for up to 6 h.

Although an established healthy reference range for plasma creatine does not exist in dogs, we attempted to define one, 33.6–132.4 μmol/L, noting that this is limited to only eight dogs of the same age and breed. Different serum creatinine reference ranges have been defined for different breeds of dogs; 70–150 μmol/L in healthy mixed breed dogs and 106–168 μmol/L in greyhounds (10). Dogs supplemented with creatine in this trial had plasma creatinine concentrations above both reference ranges from 30 to 300 min following the meal. Furthermore, at certain time points following the meal, dogs fed CON, TAU, and MET also had concentrations that exceeded the mixed breed reference range. Although this reference range is in serum and we measured plasma, this should be considered when using creatinine as a diagnostic tool for kidney disease in dogs. In fact, in several case reports in human medicine, people consuming large amounts of protein and creatine supplements have been misdiagnosed with kidney disease due to elevated levels of serum creatinine (21). Based on the data from the present study, it is recommended that fasted blood samples be collected when assessing creatinine concentrations in dogs in a clinical setting, especially if the dog is consuming supplementary creatine, as these values could be elevated and additional diagnostics are required.

In the only study to investigate a source of creatine (guanidinoacetic acid, GAA) supplementation to Foxhound mixed breed dogs undergoing a light exercise regimen, Dobenecker and Braun (22) reported greater plasma creatine concentrations up to 7 h post-meal, as well as a decrease in body fat and an increase in muscle. This avenue of research remains largely unexplored in dogs, but supplementing creatine to human athletes has been well-studied and suggests that creatine supplementation can improve both physical performance (23, 24) and recovery after short-term, high intensity exercise (25, 26). The meal response of creatine appearance in plasma suggests that after only 7 days of supplementation, peak concentrations of creatine can reach up to seven times that of fasted concentrations. Although more research is needed, this data provides insight into the application of creatine supplementation in dogs and suggests that working breeds who perform shorter bouts of high intensity exercise may benefit from creatine supplementation, as has been shown to be the case in exercising humans (23–26).

Several studies in both humans (27) and rats (5, 6) suggest that creatine supplementation decreases homocysteine concentrations. Homocysteine has long been suggested as a risk factor for the development of coronary heart disease in humans [reviewed in (28)] and more recently, in dogs (29, 30). In previously published findings from this study, we reported similar plasma homocysteine and methionine concentrations in the CCC supplemented group compared to CON and TAU, despite high levels of supplemental creatine, whereas dogs supplemented with methionine had elevated plasma homocysteine and methionine concentrations from 60 to 360 min following the meal (12). There are several explanations for the lack of change in methionine and homocysteine concentrations in the CCC group. First, the control diet was formulated for dogs of all life stages and far exceeded amino acid requirements at maintenance. Brosnan et al. (31) highlight that the demand for *de novo* creatine synthesis in growing piglets is far greater than at maintenance and presents a considerable burden on methionine metabolism and this demand may not be present in the dogs on the current study. Second, the dogs used in the current study were healthy adult dogs not undergoing any exercise or immune challenge and therefore there was no additional demand for amino acids or downstream metabolites. Deminice and Jordao (32) also highlight the ability of creatine to reduce oxidative stress markers induced by exercise, suggesting a demand for creatine during exercise. We also did not see any treatment differences in plasma arginine or glycine in the previous report (12), which may be explained by this reasoning as well. Likely the oversupply of amino acids in the control diet circumvented the need for any sparing effect of creatine supplementation. Future studies may attempt to investigate the sparing effect of creatine with either growing or exercise-challenged dogs or perhaps a diet limited in methionine.

Recent work in human medicine has begun exploring creatine's role in disease. For example, patients with rheumatoid arthritis supplemented with creatine for 12 weeks had increased appendicular and total lean mass, improving muscle wasting caused by the disease (33). A study done in healthy elderly men and women reported that those supplemented with creatine for 2 weeks had improved upper body grip strength and delayed neuromuscular fatigue (34). Some work in rats suggests an anti-inflammatory effect of supplemental creatine in models of both acute and chronic induced inflammation (35, 36). However, studies done in humans remain contradictory. Although creatine supplementation in healthy athletes has led to decreases in several pro-inflammatory cytokines after exercise (37, 38), supplemental creatine in patients with osteoarthritis had no effect on pro-inflammatory compounds compared to unsupplemented patients (39). Together, this work suggests a possible application for creatine supplementation in aging dogs, but research is yet to be done in this area.

In conclusion, 7 days of creatine monohydrate supplementation at 9.6 g/kg DM (200 mg/kg BW/day) fed to healthy adult beagles led to elevated plasma creatine concentrations up to at least 6 h post-meal and elevated plasma creatinine concentrations up to 4 h post-meal. Although our previous findings suggest no differences in plasma methionine, arginine or glycine in the CCC group, rat and human studies indicate that this may be a possibility and may be relevant for future investigations. For instance, exercising dogs or puppies may be favorable populations of dogs to benefit from elevated energy stores or spare precursor amino acids necessary for growth. These populations should be considered when thinking about supplementing creatine in dogs and when conducting future research.

## Data availability statement

The raw data supporting the conclusions of this article will be made available by the authors, without undue reservation.

## Ethics statement

The animal study was reviewed and approved by Animal Care Committee, University of Guelph.

## Author contributions

SB and AS were responsible for designing the study. SB was responsible for statistical analysis and writing of the manuscript. UB and ES were responsible for laboratory analysis. All authors contributed to editing of the manuscript.

## Funding

This research was funded by Rolf C. Hagen, Inc. (Grant: 053974) and awarded to AS. The funders had no role in study design, data collection and analysis, decision to publish, or preparation of the manuscript.

## Conflict of interest

Author AS is the Champion Petfoods Chair in Canine and Feline Nutrition, Physiology and Metabolism, a Champion Petfoods consultant, receives research funding from private industry, and was a former employee of P&G Petcare and Mars Petcare. Author UB is a former employee of AlzChem Group AG. The remaining authors declare that the research was conducted in the absence of any commercial or financial relationships that could be construed as a potential conflict of interest.

## Publisher's note

All claims expressed in this article are solely those of the authors and do not necessarily represent those of their affiliated organizations, or those of the publisher, the editors and the reviewers. Any product that may be evaluated in this article, or claim that may be made by its manufacturer, is not guaranteed or endorsed by the publisher.
