# Supplementary material for: Addition of a combination of creatine, carnitine, and choline to a commercial diet increases postprandial plasma creatine and creatinine concentrations in adult dogs
Source: Front Vet Sci. 2022 Nov 25;9:1063169. doi: 10.3389/fvets.2022.1063169 (PMC9731106; doi:10.3389/fvets.2022.1063169)
Supplement: Supplementary file 1 [file Table_1.DOCX]

**Supplementary Table 1.** Analyzed nutrient composition of the control diet (Nutrience Grain-Free Pork, Lamb and Duck Formula, Rolf C. Hagen Inc., QC, Canada) (CON) and three treatment diets after supplementation of methionine (MET), taurine (TAU) and creatine, carnitine and choline (CCC). Values are presented on an as fed basis.

| Nutrient | CON | MET | TAU | CCC |
| --- | --- | --- | --- | --- |
| Proximate analysis (%) |  |  |  |  |
| Moisture | 6.93 |  |  |  |
| Crude Protein | 29.60 |  |  |  |
| Fat | 16.63 |  |  |  |
| Crude Fiber | 3.31 |  |  |  |
| Nitrogen-free extract (calculated) | 34.55 |  |  |  |
| Ash | 8.98 |  |  |  |
| Calculated metabolizable energy^1^ (kcal/kg) | 3659 |  |  |  |
| Indispensable amino acids (%) |  |  |  |  |
| Arginine | 2.06 |  |  |  |
| Histidine | 0.61 |  |  |  |
| Isoleucine | 1.04 |  |  |  |
| Leucine | 1.97 |  |  |  |
| Lysine | 1.77 |  |  |  |
| Methionine | 0.43 | 0.67 |  |  |
| Cystine | 0.31 |  |  |  |
| Phenylalanine | 1.15 |  |  |  |
| Threonine | 0.83 |  |  |  |
| Tryptophan | 0.23 |  |  |  |
| Valine | 1.37 |  |  |  |
| Dispensable amino acids (%) |  |  |  |  |
| Alanine | 1.64 |  |  |  |
| Aspartic acid | 2.62 |  |  |  |
| Glutamic acid | 4.03 |  |  |  |
| Glycine | 2.32 |  |  |  |
| Proline | 1.67 |  |  |  |
| Serine | 1.24 |  |  |  |
| Taurine | 0.08 |  | 0.15 |  |
| Tyrosine | 0.76 |  |  |  |
| Other (mg/kg) |  |  |  |  |
| Choline | 2393 |  |  | 3534 |
| L-carnitine | 81 |  |  | 307 |
| Creatine | 195 |  |  | 8928 |
| Creatinine | 563 |  |  |  |
